# Katanin-p80 Gene Promoter Characterization and Regulation via Elk1

**DOI:** 10.1371/journal.pone.0069423

**Published:** 2013-07-24

**Authors:** Ece Selçuk, Koray Kırımtay, Derya Canbaz, Güher Işık Cesur, Sirin Korulu, Arzu Karabay

**Affiliations:** Department of Molecular Biology and Genetics, Istanbul Technical University, Istanbul, Turkey; University of Florida, United States of America

## Abstract

Katanin is an ATPase family member protein that participates in microtubule severing. It has heterodimeric structure consisting of 60 kDa (katanin-p60) and 80 kDa (katanin-p80) subunits encoded by *KATNA1* and *KATNB1* genes, respectively. Katanin-p60 has the enzymatic activity for microtubule severing, whereas katanin-p80 consists of multiple domains with different functions such as targeting katanin-p60 to the centrosome, augmenting microtubule severing by katanin-p60, and even suppressing microtubule severing. Despite the various important functions of katanin-p80, its transcriptional regulation has not been studied yet. Elk1 transcription factor has been shown to interact with microtubules and regulate the transcription of another microtubule severing protein, spastin. In spite of katanin’s importance, and structural and functional similarities to spastin, there is no study on the transcriptional regulation of katanin yet. In this study, we aimed to characterize *KATNB1* promoter and analyze the effects of Elk1 on katanin-p80 expression. We identified a 518- bp TATA-less promoter including a critical CpG island and GC boxes as an optimal promoter, and sequential deletion of CpG island and the GC elements gradually decreased the *KATNB1* promoter activity. In addition, we showed Elk1 binding on the *KATNB1* promoter by EMSA. We found that Elk1 activated *KATNB1* promoter, and increased both mRNA and protein levels of katanin-p80 in SH-SY5Y cells. On the other hand, KCl treatment increasing SUMOylation decreased *KATNB1* promoter activity. Since microtubule severing is an important cellular mechanism of which malfunctions result in serious diseases such as spastic paraplegia, Alzheimer’s disease and cell cycle related disorders, identification of *KATNB1* transcriptional regulation is crucial in understanding the coordination of microtubule severing activity by different proteins in the cells.

## Introduction

**Figure 1:**
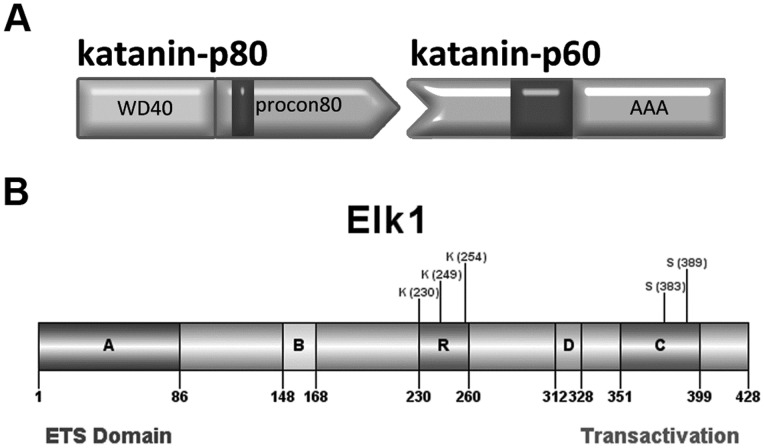
Schematic illustration of human katanin-p80 and Elk1 proteins. (**A**) Katanin-p80 has WD40 domain which negatively regulates microtubule severing activity of katanin-p60, and procon80 domain which is required for interaction with katanin-p60 and includes microtubule binding domain (dark grey). (**B**) The A domain (or ETS domain) is responsible for binding to DNA. The B domain is involved in interaction of Elk1 with SRF. The R domain including the lysine residues of Elk1 associated with SUMO modification that is important for repressive activity of Elk1. The D domain is docking site for MAP kinases. The C domain (or transactivation domain) contains serine residues at positions 383 and 389 which are phosphorylated by MAP kinases. Illustrations were performed by the use of DOG 1.0 and Adobe Photoshop CS5 softwares.

Microtubule severing is one of the important events in the regulation of microtubule dynamics which is essential for fundamental cellular processes such as cell division and differentiation. Microtubule severing is performed by enzymes such as katanin and spastin [1], [2]. Katanin is the most well characterized severing protein composed of 60 kDa (katanin-p60) and 80 kDa (katanin-p80) subunits encoded by *KATNA1* (NM_007044) and *KATNB1* (NM_005886) genes, respectively. Katanin-p60 contains a C-terminal AAA (ATPases Associated with various cellular Activities) domain which has an enzymatic activity that breaks microtubules while katanin-p80 has multiple domains (Figure 1A) with different functions. The N-terminal WD40 repeat domain of katanin-p80 is a negative regulator of microtubule severing by katanin-p60 and is necessary for centrosome or spindle pole targeting; while procon80 domain includes microtubule binding motif and is required for interaction with katanin-p60 and augmentation of its enzymatic activity [3], [4], [5].

Although both katanin-p80 and katanin-p60 are widely distributed throughout the cell, their levels have been found to change independently in different tissue types and developmental stages depending on the demand of microtubule severing. For instance, katanin-p60/katanin-p80 ratio has been less in neuronal tissues, indicating an increase in katanin-p80 level, compared to non-neuronal tissues [6]. Another study performed in primary hippocampal neurons showed that overexpression of katanin-p60 decreased process numbers, whereas overexpression of katanin-p80 elevated process numbers probably by changing configuration of microtubule array to promote the formation of more processes, presumably by enhancing release of microtubules from the centrosome or increasing the rate of exodus of microtubules from the cell body [6].All these findings indicate that katanin-p80 contributes to the efficacy of katanin-p60 by increasing its severing efficiency, indicating that katanin-p80 has likely other roles in addition to targeting katanin-p60 to the centrosomes [5].

The microtubule severing mechanisms of katanin and spastin have been intensively studied for many years. Yet, the underlying reasons for their different modes of action and distribution still remain to be solved. For example, although both katanin and spastin are members of the same ATPase family and severe microtubules, each has a unique structure such that katanin is a dimer, whereas spastin is a monomer [1], [2]. While both spastin and katanin are expressed in cells simultaneously, one cannot compensate any loss of function of the other [7]. In addition to this, severing by katanin is only possible when microtubule associated proteins (MAP) detach from microtubules, whereas severing by spastin is independent of whether or not MAP remain attached to the microtubules [8]. Therefore, the coordination of these seemingly redundant, but non-compensable severing proteins’ functions requires extensive regulations to keep the cells from detrimental effects of excessive severing by these proteins. The alternative severing by these proteins could be achieved at the transcriptional level by the action of different transcription factors. Among these transcription factors, Elk1’s (E twenty-six (ETS)-like transcription factor 1) regulation has already been studied on the spastin promoter, and it has been shown to negatively regulate the transcription of spastin [6].

In the putative transcription factor binding sites searched for the presence on the *KATNB1* promoter region also resides an Elk1 binding site. Besides its function as a transcription factor, Elk 1 has distinct cellular roles proven by its presence in non-nuclear compartments [9], [10]. Phosphorylated Elk1 is localized in the nucleus of the cells and functions as a transcription factor. However, in its unstimulated state, it becomes cytoplasmic and interacts with microtubules in primary hippocampal neurons [11]. This non-nuclear localization of Elk1 also coincides with its distinct cellular roles in neurons such as regulation of synaptic plasticity and dendrite elongation [9], [10]. Elk1 is composed of several domains with different functions (Figure 1B), and it can either activate or repress the expression of the target gene [12]. The activity of Elk1 is regulated by phosphorylation/dephosphorylation or SUMOylation events depending on the type of cellular signaling. MAP kinase mediated phosphorylation of Elk1 stimulates transcriptional activity by Elk1 through pathways such as ERK (ERK1 and ERK2; extracellular signal regulated kinase), JNK (c-Jun N-terminal kinase) and p38 cascades [13]. On the other hand SUMO (Small Ubiquitin-like Modifier) modification of Elk1 on the R motif represses the transcription of the target gene [14].

Elk1’s property of bearing different functions depending on its post-translational modification state, and as a result, possible different effects on the target gene of interest made this protein an important candidate to be studied in the transcriptional regulation of katanin-p80. In addition, protein kinase cascades play critical roles in the signaling events that underlie synaptic plasticity, and one of the proteins involved in synaptic plasticity by affecting microtubule network is spastin [15] which has already been shown to be regulated by Elk1 [7]. Disruption of synaptic form and function occurs comparatively early, preceding the onset of degenerative changes in the neuronal cell body [16] and neurodegeneration has recently been shown to extend to katanin-related microtubule disruption [17]. There has been an increasing awareness on the importance of transcriptional regulation of spastin [18,19,20 and 21]. Yet, in spite of katanin’s importance, and structural and functional similarities to spastin, there is no study on the transcriptional regulation of neither katanin-p60 nor katanin-p80 up to date. Multifunctional structure and diverse roles of katanin-p80 on the regulation of microtubule severing by katanin-p60 made it an important subunit of the katanin for the promoter characterization and transcriptional regulation studies. Based on the roles of Elk1 and spastin in synaptic plasticity and similarity of katanin’s to spastin, we aimed to characterize the *KATNB1* promoter and identify the regulatory roles of Elk1 transcription factor on katanin-p80 expression.

For this purpose, the first step was to characterize the katanin-p80 promoter, and to do that we obtained deletion constructs for the predicted promoter region. 518- bp region (between -2013 and -1496) was identified as having the highest promoter activity and 160- bp region (between -1655 and -1496) was found to act as a minimal promoter for the *KATNB1* gene. Moreover, Elk1’s binding to its putative binding site within the *KATNB1* promoter was confirmed. Upon binding, Elk1 activated *KATNB1* promoter and increased both mRNA and protein levels of katanin-p80 in SH-SY5Y human neuroblastoma cells. In addition, we triggered SUMO conjugation to Elk1 with KCl treatment, which is known to increase SUMOylation [22] and found that SUMOylated Elk1 repressed *KATNB1* gene expression.

## Results

### *KATNB1* Gene Promoter is Composed of 518- bp Optimal Promoter and 160- bp Minimal Promoter

**Figure 2:**
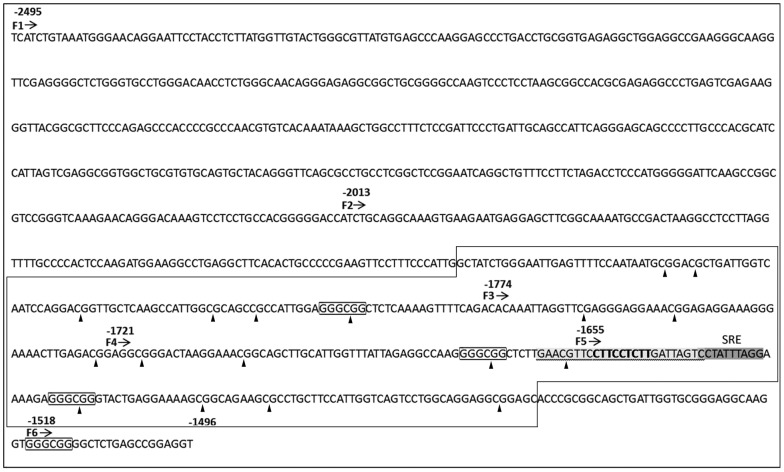
Putative Elk1 transcription factor binding site on the *KATNB1* promoter. Forward primer binding sites for each construct are indicated as F1, F2, F3, F4, F5 and F6. Elk1 binding site represented as bold. Underlined and grey highlighted region show the probe selected for EMSA assay, the dark grey highlighted region represents serum response element (SRE) to which serum response factor (SRF) binds and GC boxes are shown in frames. The largest frame represents CpG island. CpG dinucleotides are shown with arrowheads.

**Figure 3:**
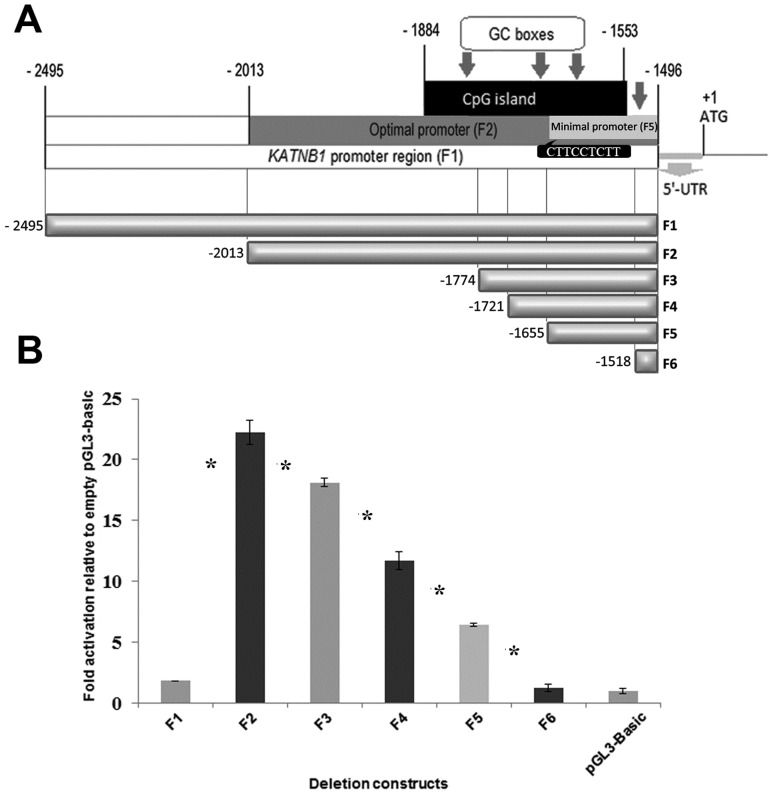
Functional analysis of the *KATNB1* promoter region. (**A**) The analyzed *KATNB1* promoter region is 1000- bp (F1) excluding 5′-UTR. The CpG island is 332 nucleotides in length and positioned between -1884 and -1553. The optimal promoter region showing the highest promoter activity is 518- bp in length and positioned between -2013 and -1496 (F2). Location of Elk1 consensus sequence (CTTCCTCTT) is indicated in the figure (**B**) Luminometric analysis of promoter constructs. SH-SY5Y cells were co-transfected with promoter constructs and pRL-TK *Renilla* luciferase vector. Results were calculated as fold activation relative to empty pGL3-basic vector. Error bars represent ± SD. Asterisk symbol (*) indicates p-value <0.05, meaning a statistically meaningful difference.

Putative *KATNB1* gene promoter is located on chromosome 16 between *CCDC135* and *KATNB1* genes (ref. seq. NM_005886) according to NCBI Map Viewer database (http://www.ncbi.nlm.nih.gov/mapview/). The 1000- bp region of *KANTB1* gene promoter (F1) (Figures 2 and 3) that is the genomic region upstream of the transcriptional start site, excluding 5′-UTR was determined via University of California Santa Cruz (UCSC) Bioinformatics Genome Browser software (http://genome.ucsc.edu/cgi-bin/hgGateway). No TATA box or CAAT box was identified, but 4GC boxes and 1 CpG island were detected by bioinformatics analyses of the promoter (http://www.ebi.ac.uk/Tools/msa/clustalw2/; http://www.ebi.ac.uk/Tools/emboss/cpgplot/) ((Figures 2 and 3).

In order to define the promoter region that is critical for the expression of *KATNB1* gene, promoter deletion fragments were generated at the most feasible intervals according to the presence of the predicted transcription factors binding sites. We generated 6 promoter deletion fragments, and they were more closely spaced towards the transcription start site in order to determine the most critical regions within the promoter (Figures 2 and 3A).

Each fragment was cloned into pGL3-basic vector and transiently transfected into SH-SY5Y cells. The activities of promoter deletion fragments were measured via expression of the luciferase gene using luciferase assay and considered as fold of induction in respect to the activity of the empty pGL3-basic vector (Figure 3B). Each experiment was performed 6 times as triplicates to obtain statistically meaningful data.

The construct that showed the highest promoter activity was F2 and inclusion of an additional 482- bp upstream of this region (F1) led to a significant decrease in the promoter activity. Subsequent deletions of the promoter region from F2 to F3, F4, F5 and F6 regions caused continuous significant decreases in the promoter activity. Gradual removal of the CpG island with the deletion constructs (from F3 to F5: -1774 to -1655) caused a gradual decrease in the promoter activity. The subsequent deletion of the end of the CpG island (from F5 to F6: -1655 to -1518) caused a substantial decrease in the promoter activity. Therefore, the ∼ 160- bp region (located between -1655 and -1496) containing the terminal part of the CpG island (F5 construct) showed the minimal promoter activity. Deletion of the entire CpG island (F6 construct containing the region between -1518 and-1496) completely abolished the promoter activity (Figure 3A and B).

These results indicate that the 2013- bp upstream of the first ATG acts as an optimal promoter and 1655- bp upstream of the first ATG acts as a minimal promoter for *KATNB1* gene expression.

The newly identified *KATNB1* gene optimal promoter sequence (F2) (Figure 2) data has been deposited in GenBank with the accession number of KF039688.

### Identification of Elk1 Binding Site on the Optimal *KATNB1* Promoter

We theoretically searched the presence of Elk1 (AB016193) transcription factor binding sites (AC: T00250) on the *KATNB1* promoter by using PROMO bioinformatics tool [23], [24] restricting the maximum matrix dissimilarity rate 0–3% for *Homo sapiens*. Bioinformatics analysis predicted only one Elk1 binding site positioned between -1652 and -1644 in the *KATNB1* promoter (Figure 2 and 3A).

### Confirmation of Elk1 - *KATNB1* Promoter Binding

**Figure 4:**
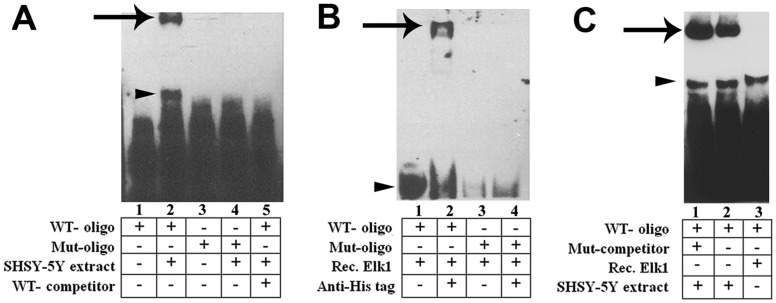
Confirmation of Elk1 binding to the corresponding site on the *KATNB1* promoter by EMSA. (**A**) Extract free biotin-11-UTP labeled Elk1 oligonucleotides (WT, wild type; Mut, mutated) were illustrated in lanes 1 and 3, respectively. SH-SY5Y cell extract was added to the reaction mixture and incubated with WT and Mut oligonucleotides to form nucleotide-protein complex (lane 2 and lane 4, respectively). 1000-fold excess of unlabeled WT competitor Elk1 oligonucleotide was added to the binding reaction mixture (lane 5). Arrow shows band of SH-SY5Y cell extract-WT oligonucleotide complex (lane 2). Lane 1 and 3 represent only WT and Mut oligonucleotides, respectively that were loaded as controls. (**B**) Super-shift assay confirmed the specificity of Elk1 binding on the *KATNB1* promoter. WT Elk1 oligonucleotide was incubated with recombinant Elk1- db protein either alone (lane 1) or in the presence of His-tag antibody (lane 2). Mut Elk1 oligonucleotides were also incubated with recombinant Elk1- db protein either alone (lane 3) or in the presence of His-tag antibody (lane 4). Arrowhead shows band of Elk1- db-WT oligonucleotide complex and the arrow shows the band of Elk1- db-WT oligonucleotide-His-tag antibody complex. (**C**) SH-SY5Y cell extract was added to the reaction mixture and incubated with WT oligonucleotides in the presence or absence of 1000 fold excess of unlabeled Mut competitor oligonucleotides to form nucleotide-protein complex (lane 1 and lane 2, respectively). Lane 3 shows Elk1- db-WT oligonucleotide complex. Arrows show bands of SH-SY5Y cell extract-WT oligonucleotide complexes and arrowheads indicate 95 amino acids splice variant of Elk1.

The binding ability of Elk1 transcription factor to the theoretically predicted Elk1 binding site on the *KATNB1* promoter was confirmed by EMSA (Figure 4).

For this purpose, we used wild type (WT) and mutated (Mut) oligonucleotides that contain the wild type consensus and mutated Elk1 DNA-binding sequences, respectively (Table 1). The Elk1 binding was tested in the presence of WT, Mut and competitor oligonucleotides. Results confirmed Elk1’s binding to the *KATNB1* promoter as indicated with an arrow in Figure 4A, lane 2.

**Table 1:** Oligonucleotides used in EMSA.

Oligo name	Sequence
Elk1_WT	5′-GAACGTTCCTTCCTCTTGATTAGTC-3′
Elk1_Mut	5′- GAACGTTCTCCTTCTCCGATTAGTC -3′

Oligonucleotide containing Elk1 binding site on *KATNB1* promoter underlined and named as Elk1_WT. Purine-purine and pyrimidine-pyrimidine converted oligonucleotide corresponding to Elk1 binding site named as Elk1_Mut.

In the absence of cell extracts, no binding was observed (Figure 4A, lanes 1 and 3). Both Mut and competitor oligonucleotides inhibited the binding, as expected (Figure 4A, lanes 4 and 5, respectively).

In order to further confirm specifically the binding of Elk1 to the binding site, we used the 6XHis tagged Elk1 recombinant protein (Elk1- db) containing only the DNA binding domain of WT Elk1 [7]. WT and Mut oligonucleotides were incubated in the presence of Elk1- db specifically, instead of total cell extracts. In order to observe super-shifted bands that would indicate the specificity of Elk1- db - *KATNB1* promoter binding, gel was over-electrophoresed (Figure 4B). The complex formation of Elk1- db with the binding site was observed in the presence of WT oligonucleotides (arrowhead in Figure 4B, lane 1) at the bottom of the gel as it was over-electrophoresed. The complex formation was prevented in the presence of Mut oligonucleotides (arrowhead in Figure 4B, lane 3).

In these super-shift experiments, anti-His tag antibody was used to ensure that the protein bound to *KATNB1* promoter was Elk1-db. Using anti-His tag antibody, the super-shifted complex was shown to contain the 6XHis tagged Elk1 recombinant protein (Elk1-db) (Figure 4B, lane 2, arrow). The super-shifted complex was abolished when the residues in the binding site were mutated (arrow in Figure 4B, lane 4).

Since we observed an additional binding (indicated with arrowhead in Figure 4A, lane 2) that was absent in the cell extracts with the labeled mutant (indicated with arrowhead in Figure 4A, lane 4) and unlabeled WT-competitor (indicated with arrowhead in Figure 4A, lane 5) containing complexes, we further tested the specificity of this binding by using unlabeled mutant competitor (1000 fold excess of WT oligonucleotides) and WT-oligonucleotides (Figure 4C, lane 1). This additional binding was again observed in both cell extracts and Elk1 recombinant protein (Elk1-db) in the presence of mutant competitor and/or WT oligonucleotides (arrowhead in Figure 4C, lanes 1, 2, 3,), indicating the specificity of the binding. The cell extract samples showed the presence of a higher signal shift as indicated with the arrow in Figure 4C, lanes 1, 2, and that higher signal shift was absent in the absence of cell extract (arrow in Figure 4C, lane 3).

### Elk1 Activated *KATNB1* Promoter and Increased Katanin-p80 mRNA Level

**Figure 5:**
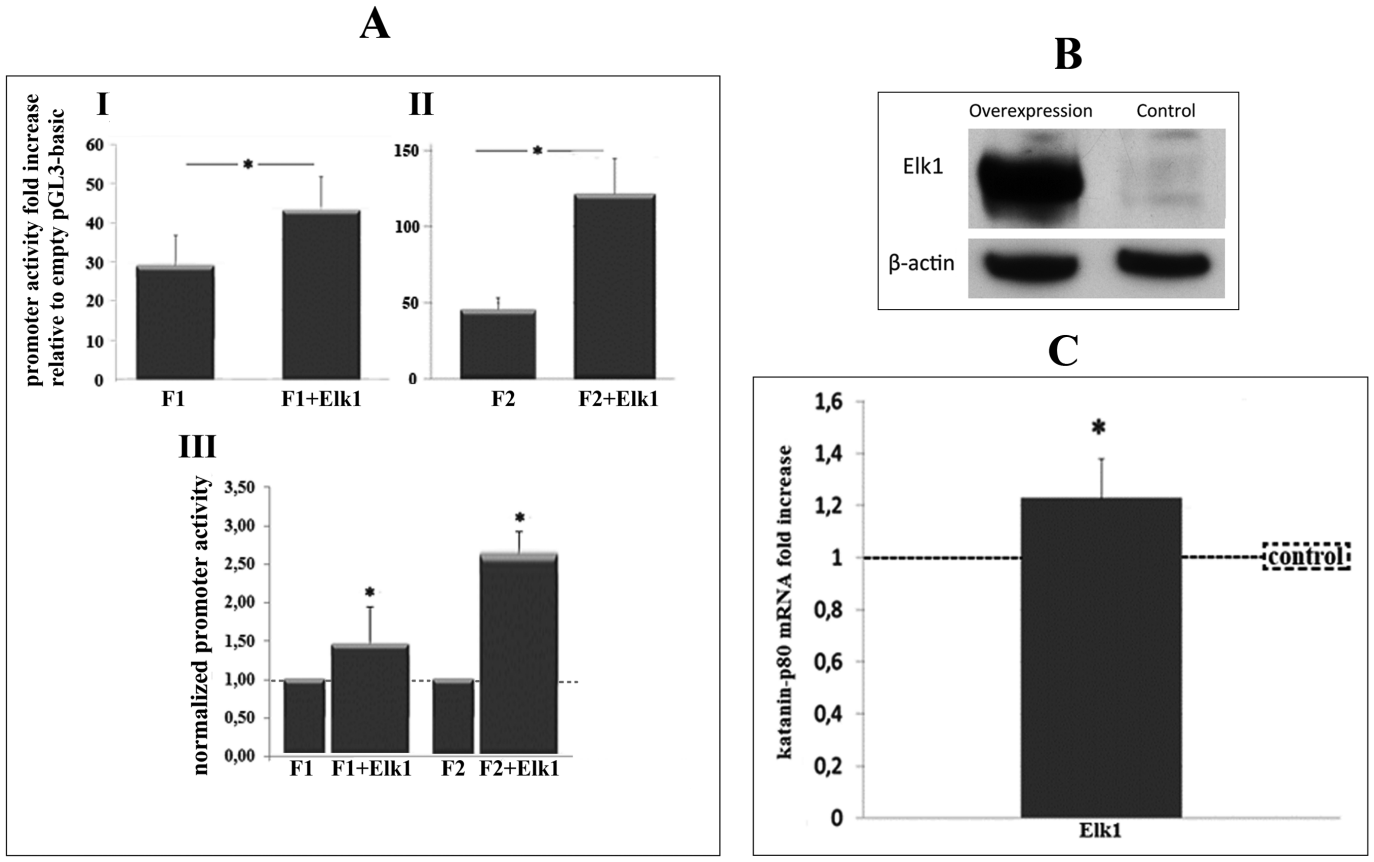
Elk1 activates KATNB1 promoter and increases katanin-p80 mRNA. (**A**) Forced luciferase experiment to compare effects of Elk1 on KATNB1 optimal promoter activation. SH-SY5Y cells were co-transfected with (I) F1 construct and either pCMV-empty vector (F1) or pCMV6-Elk1 (F1+Elk1) and (II) F2 construct and either control (F2) or Elk1 (F2+Elk1). (III) Normalization of data is shown in I and II. Promoter activity of F1 and F2 constructs transfected with Elk1 containing vector were normalized to F1 and F2 constructs transfected with control vector, respectively. (**B**) Western blot image represents the total protein lysate obtained either from Elk1 transfected (overexpression) or naive SH-SY5Y cells (control). Cells were immunolabeled for Elk 1 and β-actin. (**C**) Relative mRNA quantification of katanin-p80 mRNA level in Elk1 overexpressing SH-SY5Y cells (Elk1). Results were calculated by normalizing the Ct values of the KATNB1 gene to β-actin as internal control. Results represent ΔΔCt values which gives mRNA fold change relative to naive cells (control). Error bars represent ± SD. Asterisk symbol (*) indicates p-value <0.05, meaning a statistically meaningful difference.

After confirming Elk1’s ability to bind its predicted binding site by EMSA, in order to identify the effects of this transcription factor on the promoter, SH-SY5Y cells were co-transfected with Elk1 transcription factor in the presence of either F1 or F2 construct by a procedure named “forced experiment” (Figure 5).

Transfection experiment was performed as triplicates on the same day and the experiment was replicated 3 times on separate days independently in order to have statistically meaningful data.

In order to understand if Elk1 could overcome the repressive effect present in F1 region, SH-SY5Y cells were co-transfected with either F1 construct and pCMV-empty vector (F1) or F1 construct and pCMV6-Elk1 vector (F1+Elk1) (Figure 5A, I).

SH-SY5Y cells were also co-transfected with either F2 construct (optimal promoter) and pCMV-empty vector (F2) or F2 construct (optimal promoter) and pCMV6-Elk1 vector (F2+Elk1) (Figure 5A, II).

Luciferase data showed that Elk1 increased promoter activity of both F1 and F2 constructs compared to control conditions (Figure 5A, I and II, respectively). When Elk1 forced results were normalized to control results (Figure 5A, III), it was observed that Elk1 enhanced promoter activity 1.47-fold in F1 and 2.64-fold in F2 construct.

After confirming Elk1 could actually activate katanin-p80 promoter, in order to further quantify whether Elk1’s binding to the katanin-p80 promoter would functionally reflect to any change at the mRNA level of katanin-p80, we performed qRT-PCR. In order to directly correlate if any possible change in katanin-p80 mRNA level could actually be based on Elk1 level, we first showed the overexpression of Elk1 in SH-SY5Y cells (Figure 5B). For this purpose, the basal and overexpressed levels of Elk1 were determined by performing Western blot analysis in both naive (control-basal level) and pCMV6-Elk1 transfected (overexpressed) SH-SY5Y cells. Western blotting results confirmed that amount of Elk1 was much higher in pCMV6-Elk1 transfected SH-SY5Y cells compared to control cells (Figure 5B).

After confirming successful expression of Elk1 in SH-SY5Y cells, total mRNA was isolated from pCMV6-Elk1 overexpressing SH-SY5Y cells and expression analysis of *KATNB1* was performed. Relative quantification analysis of qRT-PCR data revealed an increase (values above 1) in mRNA levels of katanin-p80 in Elk1 overexpressing SH-SY5Y cells indicating an up-regulation of the target gene expression (Figure 5C).

### KCl Treatment Increased Elk1 SUMOylation and Decreased *KATNB1* Gene Expression

We performed KCl treatment to the cells to induce an increase in SUMOylation [22] in order to understand whether SUMO modifications would affect Elk1 mediated increase in *KATNB1* promoter activity. For this purpose, 24 h post-transfection, Elk1 transfected cells were incubated for 1 h in 50 µM KCl containing medium. 48 h post-transfection, luminometrical measurement results were evaluated.

**Figure 6:**
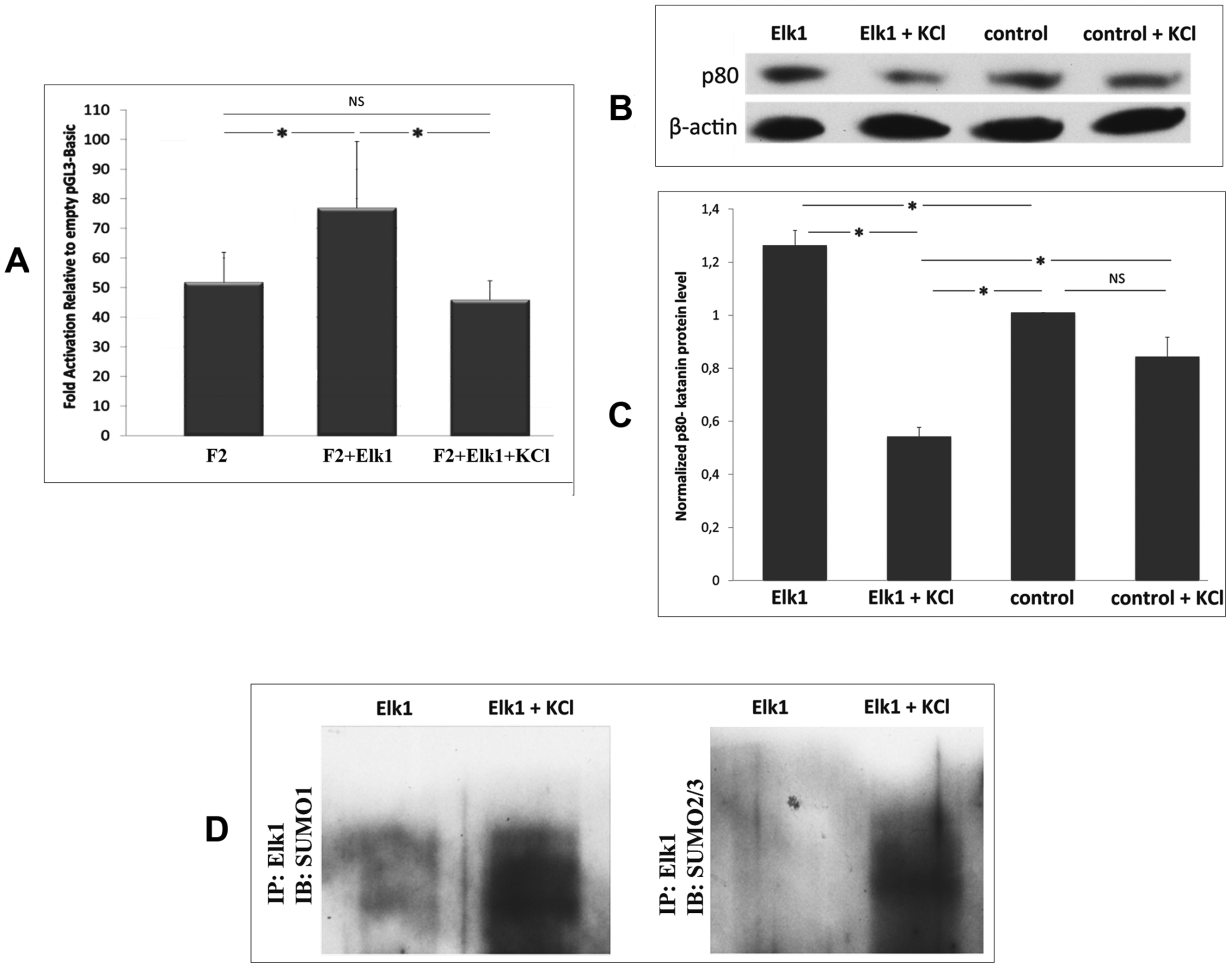
KCl treatment increases Elk1 SUMOylation and decreases katanin-p60 expression. (**A**) The effect of KCl treatment on Elk1 mediated regulation of *KATNB1* promoter. SH-SY5Y cells were co-transfected with F2 construct and either pCMV-empty vector (F2), or pCMV6-Elk1 (F2+Elk1). 24 h post-transfection, group of the Elk1 transfected cells were incubated in medium containing 50 µM KCl for 1 h (F2+Elk1+KCl). After 48 h following transfection, luminometric analysis was performed and fold activities were calculated relative to empty pGL3-basic vector. (**B**) Western blot analysis of katanin-p80 in SH-SY5Y cells. We performed analysis with both pCMV6-Elk1 transfected cells which were either untreated (Elk1) or treated with KCl (Elk1+KCl) and naive cells which were either untreated (control) or treated with KCl (control+KCl). Error bars represent ± SD. Asterisk symbol (*) indicates p-value <0.05, meaning a statistically meaningful difference and not significant (NS) symbol represents vice versa. (**C**) Quantification of Western blot image represented in panel B. Quantifications were obtained by normalizing band intensities of katanin-p80 to band intensities of β-actin. (**D**) Western blot analysis of immunoprecipitated Elk1. Total protein was extracted from untreated (Elk1) and KCl treated (Elk1+KCl) Elk1 overexpressing cells and SUMOylation levels were observed by using SUMO1 and SUMO2/3 antibodies.

We observed a significant decrease in KCl treated-Elk1 transfected cells’ (F2+Elk1+KCl) promoter activity compared to KCl untreated-Elk1 transfected cells (F2+Elk1) due to increased SUMO conjugation (Figure 6A). Compared to control cells (F2 without Elk1 force), the promoter activity was significantly increased upon Elk1 force, but the increased promoter activity was reduced significantly upon KCl treatment.

In addition to luminometrical measurement analysis, Western blotting was performed (Figure 6B) to evaluate endogenous katanin-p 80 protein levels in Elk1 transfected cells, Elk1 transfected and KCl treated cells, Elk1 untransfected (control) cells and Elk1 untransfected and KCl treated (control+KCl) cells. Quantification results were obtained by normalizing band intensities of katanin-p 80 to band intensities of β-actin (Figure 6C). Results indicated that Elk1 overexpressing cells had the highest amount of katanin-p80 protein level. On the other hand, KCl treatment causing an increase in SUMO modification, decreased katanin-p80 protein level both in Elk1 transfected and untransfected cells (Figure 6C). Furthermore, to confirm the increase in SUMOylation of Elk1, immunoprecipitation was performed. SUMO proteins were determined with both specific rabbit monoclonal anti-SUMO1 (Cell Signaling) and rabbit polyclonal anti-SUMO2/3 (Santa Cruz) antibodies. Results indicated an increase in SUMOylated Elk1 upon KCl treatment. When we compared SUMOylated Elk1 in KCl untreated and treated cells in terms of SUMO isoforms, it was obvious that Elk1 was SUMOylated with SUMO-1 rather than SUMO-2/3 in untreated cells. However, upon KCl treatment, SUMO conjugation levels were about the same with both SUMO1 and SUMO2/3 isoforms (Figure 6D).

**Figure 7:**
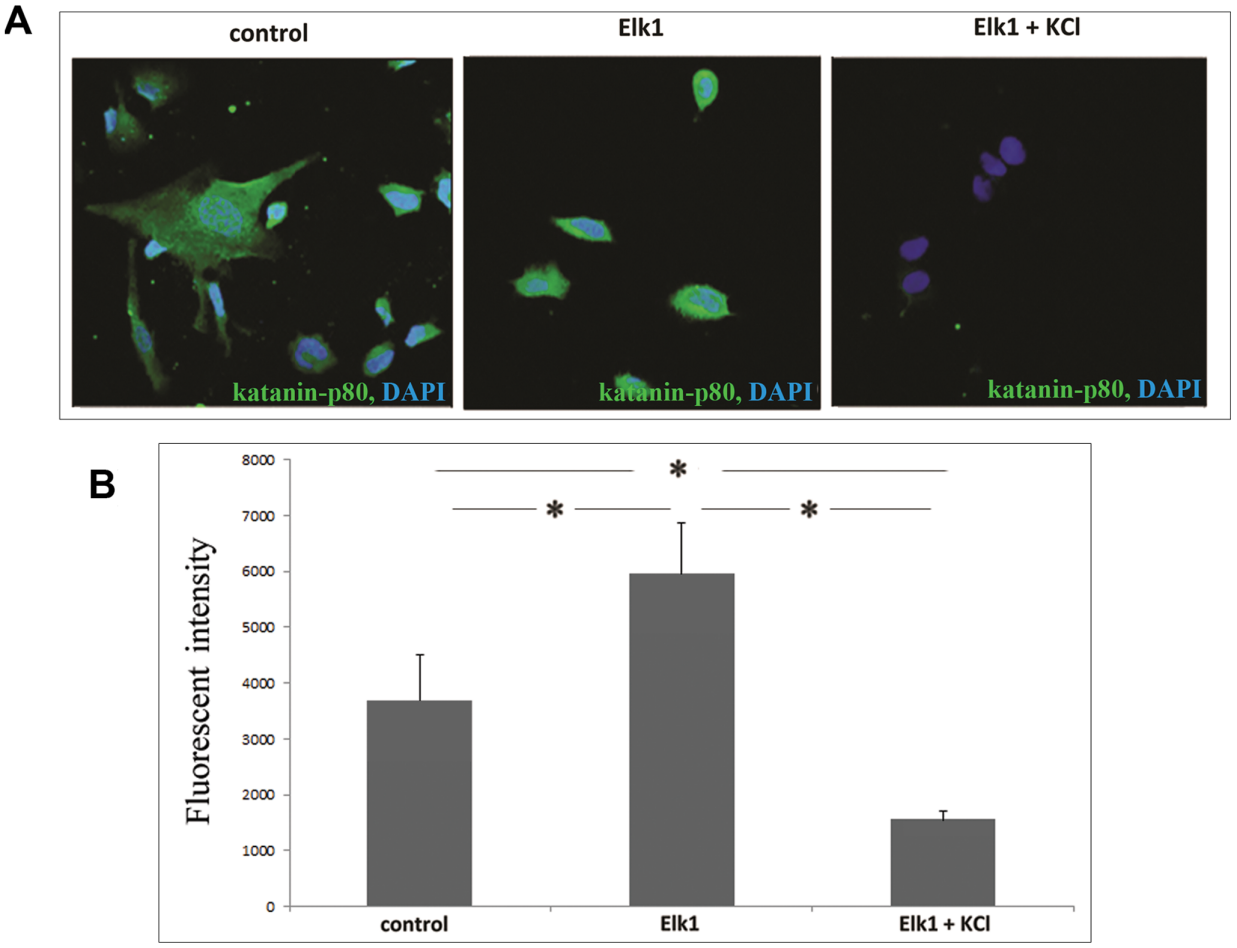
KCl stimulated Elk1 SUMOylation reduces endogenous katanin-p60 protein level in SH-SY5Y cells. (**A**) Immunocytochemistry staining of endogenous katanin-p 80 to reveal the effect of KCl treatment on the Elk1 mediated expression of *KATNB1* gene in SH-SY5Y cells. Control represents naive SH-SY5Y cells that were neither transfected nor KCl treated; Elk1 represents SH-SY5Y cells that were pCMV6-Elk1 transfected and finally Elk1+KCl represents SH-SY5Y cells that were both pCMV6-Elk1 transfected and KCl treated. Green indicates katanin-p80 staining, while blue is for nuclear DAPI staining. (**B**) Integrated pixel analysis for katanin-p80 expression in panel A.

Lastly, immunocytochemistry was performed to observe and analyze cells for changes in the protein expression level of katanin-p 80. 48 h following transfection, SH-SY5Y cells were fixed with cold methanol and prepared for immunofluorescence staining as described in detail in methods section. In concordance with Western blotting results, morphological and quantitative analysis of immunocytochemistry results revealed that Elk1 overexpressing cells increased katanin-p80 levels compared to control cells which are naive SH-SY5Y cells. On the other hand, Elk1 overexpressing and KCl treated cells contained lower katanin-p80 levels compared to both control and Elk1 overexpressing cells (Figure 7A). KCl treatment reversed Elk1’s activator effects on *KATNB1* promoter and decreased katanin-p80 expression as confirmed with integrated pixel analysis (Figure 7B). All these data show that KCl treatment triggers an increase in SUMO conjugation and SUMOylated Elk1 represses the expression of endogenous katanin-p80 protein in SH-SYSY cells.

## Discussion

The major aims of this study were to identify the optimal promoter region of *KATNB1* gene and to investigate the regulation of *KATNB1* gene expression by Elk1 transcription factor.

For the promoter characterization, the deletion constructs of *KATNB1* promoter region were designed based on the presence of transcription factor binding sites and GC rich regions, since these sites and regions have important roles in the formation of transcription initiation complex [25]. Bioinformatics results did not reveal any functional TATA or CAAT boxes on the promoter region of *KATNB1* gene which are critical sites for transcriptional regulation. When the TATA-less putative promoter was analyzed, it was shown that deletion construct F2 had the highest promoter activity. F2 includes the entire CpG island, all 4 GC-boxes, and critical transcription factor binding sites that would regulate *KATNB1* promoter. On the other hand, promoter activity of the cells induced by promoter construct F1 was not as high as the cells’ promoter activity induced by F2. The explanation of this reduced activity could be that 482 bp upstream region (spanning the region from -2495 to -2013) of F2 construct might contain possible inhibitor binding sites. On the other hand, cells that were induced with minimal promoter (F5) construct which included the terminal part of the CpG island and 2 GC boxes had lower promoter activity. Whereas, F6 construct which included a single GC box and no part of the CpG island showed almost no promoter activity, and it was about the same as basal level promoter activity. Therefore, the decrease in the number of GC boxes with the shortening constructs was associated with the decrease in the promoter activity (Figure 3A and B). GC boxes are the regulatory elements that are widely distributed in promoters of genes and may contribute tissue specific expression by GC box binding transcription factors in response to diverse stimuli [23]. In the light of these data, GC boxes localized on *KATNB1* promoter had functional roles in the activation of the promoter, and acted as positive regulatory elements. In addition to the sequential loss of GC boxes, the decrease in the promoter activity could probably be related to the loss of possible activatory transcription factor binding sites within these deletion constructs. Furthermore, addition of 110 bp to the upstream of F3 (the region between -1884 and - 1775) might have had the promoter activity as F2, as it would also include the entire CpG island and all 4 GC boxes. Besides GC boxes, the CpG island which is an important regulatory region was shown to be critical for the *KATNB1* promoter, as well. Elk1 is one of the transcription factors that could bind to corresponding sequence within the CpG island.

Elk1 transcription factor binding to *KATNB1* promoter was confirmed by EMSA as seen that Elk1-DNA complex migrated more slowly than the free DNA and mutation of the binding sites prevented the complex formation. EMSA results also indicated that the additional binding observed (Figure 4A, lane 2 arrowhead) was specific for cell extract (Figure 4A and 4C) and Elk1-db recombinant protein (Figure 4C). The close proximity of the migrated bands within the cell extracts (Figure 4C, lanes 1 and 2) and recombinant Elk1-db (Figure 4C, lane 3) indicates the presence of another form of Elk1 in the cell extract. While we were working on Elk1 binding to *KATNB1* promoter, a recent deposition was made in the database (NCBI) on April 17, 2013. This deposition indicates the presence of a splice variant form of Elk1 (splice variant 3, 95 amino acids, N terminal DNA binding domain, NP_001244097.1). The molecular weight of the complex is very similar to the complex including recombinant Elk1-db (96 amino acids) – WT oligonucleotide complex. The slight shift between the N terminal DNA binding domain including splice variant (95 amino acids) and the recombinant Elk1-db (96 amino acids) is also as a result of 6XHis tag attached to the recombinant Elk1-db. These data confirm that both full length Elk1 and Elk1-db splice variant could actually bind to the predicted binding region on the *KATNB1* promoter, once again proving the specificity of our recombinant Elk1-db to the *KATNB1* promoter.

Binding of Elk1 to both F1 and F2 promoter constructs increased the promoter activity. Yet, the increase in F1 promoter region (∼1.5 fold) was not as high as the increase in F2 promoter region (∼2.5 fold). It is possible that the region from -2495 to -2013 (from F1 to F2) contains negative regulatory elements. Although both F1 and F2 promoter constructs contain the Elk1 binding site, some of the transcription factors binding sites that are present in F1 are not included in F2 promoter region. Based on our research, we know that YY1 transcription factor negatively regulates the *KATNB1* promoter (unpublished data). YY1 transcription factor is known to negatively regulate the activities of many genes [26,27 and 28] and the majority of YY1 binding sites in *KATNB1* promoter are located in F1’s -2495 to -2013 region that is not included within the F2 construct. This region (482 bp) of F1 is highly conserved (∼%100) within the primates, and it is not conserved in other organisms, whereas F2 region is more conserved among different types of organisms. The F1 promoter region of human includes binding sites for transcription factors such as FOXP3, NF-1, RXR-alpha and RAR-beta that are mainly involved in differentiation, and these binding sites are absent in F2 region of the *KATNB1* promoter.

Expression analysis revealed that Elk1 acted as an activator on katanin-p80 promoter, since we observed elevated mRNA levels in Elk1 overexpressing cells compared to control cells. Elk1 increased the activity of the optimal promoter about 2.5 fold in luciferase experiments (Figure 5A-III, F2+Elk1) whereas it caused about 1.2 fold increase in mRNA expression of katanin-p80 (Figure 5C). Due to the nature of the forced luciferase experiments, the optimal promoter (F2) construct was introduced into the cells in a vector. Yet, Elk1 transfected SH-SY5Y cells used in RT-PCR analysis to determine the mRNA level contain the 1000- bp region intrinsically and this region corresponds to F1 construct which includes the inhibitory regulatory elements. Therefore, these elements would be likely to cause a decreased level of mRNA transcription intrinsically in SH-SY5Y cells. The level of increase in katanin-p80 mRNA level was consistent with the level of increase in katanin-p80 protein level which was also about 1.2 fold (Figure 6C, Elk1).

In contrast with its activator role on *KATNB1* promoter, our group has previously shown that Elk1 has a repressive role on *SPG4* gene promoter [7]. The reason of this opposite Elk1 activity is probably due to SRE (serum response element) sequence which is found only in *KATNB1* promoter, but not on *SPG4* promoter. SRE consensus sequence CC(A/T)_6_GG resides at position from -1636 to -1627, at the juxtaposition of Elk1 binding site (Figure 2). Thus, Elk1 could associate with SRF (serum response factor) which would bind to SRE. This interaction could result in ternary complex formation and stimulate *KATNB1* gene expression. Furthermore, both Elk1 and SRF are usually activated by MAPK pathway. Stimulation of them by the same pathway might provide cooperation of them on the target gene promoter.

Elk1 is known to undergo two different protein modifications. It is either phosphorylated by MAPK or SUMOylated [14]. Phosphorylated Elk1 usually acts as an activator, whereas SUMOylated Elk1 acts as a repressor on the promoter of the target gene [14]. There are 4 SUMO isoforms identified so far in human, SUMO-1, SUMO-2, SUMO-3 and SUMO-4. Among these isoforms, SUMO-2 and SUMO-3 are 95% homologous at the protein level, while SUMO-1 shows 47% homology with SUMO-2/3 [24]. Since SUMO-4 has been shown to have restricted pattern of expression with the highest level in the kidney [29] we mainly focused on SUMO-1 and SUMO-2/3 (Figure 6D) in our study performed in SH-SY5Y cells. Here, Elk1’s SUMOylation was triggered by KCl treatment and it was shown that SUMOylated Elk1 acted as a repressor on *KATNB1* gene promoter. When we compared SUMOylated Elk1 in KCl untreated and treated cells in terms of SUMO isoforms, it was obvious that Elk1 was normally SUMOylated with SUMO-1 rather than SUMO-2/3 in untreated cells. However, SUMO-1 and SUMO-2/3 levels were similar in KCl treated cells, supporting the idea that Elk1 is not simultaneously modified with both SUMO-1 and SUMO-2/3 in SH-SY5Y cells in KCl untreated state.

Once Elk1 is SUMOylated, it cannot be phosphorylated and it dramatically decreases the level of target gene expression. SUMOylation brings Histone deacetylases (HDAC) to the promoter region [30], [31], and therefore SUMOylated Elk1 acts as a repressor. The repressor effect of Elk1 upon SUMOylation was confirmed with significant decreases in katanin-p80 protein level upon KCl treatment compared to Elk1 overexpressing and control cells, as seen in Western blotting and immunocytochemistry results (Figure 6B–C and 7A–B). Whereas in forced luciferase experiments, KCl treatment effect was not that significant such that we did not observe a highly significant decrease in the promoter activity of SUMOylation induced Elk1 overexpressing cells (Figure 6A, F2+Elk1+KCl) compared to control (F2). Due to the nature of the forced luciferase experiments, the promoter construct was introduced into the cells in a vector, and therefore the naked optimal *KATNB1* promoter (F2) construct would not include histones that would be the targets of HDAC. As a result, because there would not be any interaction with HDAC, the decrease is not reflected at that high level in the promoter activity measured by luminometer. Whereas in Western and immunocytochemistry experiments, overexpressed and SUMOylated Elk1 could interact with HDAC on the promoter present intrinsically in the cells, and the outcome is significantly reflected as a decrease in the endogenous katanin-p80 protein level.

In a eukaryotic gene structure, in addition to the promoter region which is typically adjacent to the upstream of a gene, there are many regulatory elements that can be found in the downstream of promoters. For instance, 5′ untranslated region (UTR) and 5′-UTR introns (5′-UI) are the regions that can influence the level of gene expression. Approximately 35% of human genes (especially the genes with regulatory roles) contain introns within the 5′-UTR [32] and katanin-p80 seems to be one of them. 5′-UI of katanin-p80 might have important regulatory functions as to control the tissue specific expression and also tissue dependent alternative splicing. Since the functions of 5′-UIs are thought to be critical, examination of katanin-p80’s 5′-UI would be important to further understand the mechanisms underlying transcriptional regulation of katanin-p80.

In conclusion, we identified the critical and optimal regions on *KATNB1* promoter. We also showed that Elk1 has an activator role on the regulation of *KATNB1* promoter. In addition, CpG island which includes the Elk1 binding site is important for the promoter activity and GC boxes play activatory role on *KATNB1* gene expression.

This study would have a great impact to understand different gene expression patterns of katanin-p80, resulting different katanin-p60/katanin-p80 ratios both in development and different tissues, causing different degree of severing by katanin-p60. Therefore, it will also give insight into understanding of functional differences in different microtubule severing proteins, katanin and spastin. Based on the critical regions present on *KATNB1* promoter, it is likely that in addition to the transcriptional regulation, CpG island methylation, as in epigenetic level regulation, could also be important for katanin-p80 expression. The epigenetic level regulation of katanin promoter awaits for further investigation in order to better understand the microtubule severing activity and its effects within cells.

## Materials and Methods

### Materials

Dulbecco’s modified Eagle’s medium (DMEM, 1 g/L glucose) and fetal bovine serum were obtained from Invitrogen Corp. (Carlsbad, CA, USA). L-Glutamine and Penicillin/Streptomycin were provided from Biochrom (Cambridge, UK). Elk1 and mutated Elk1 oligonucleotides were synthesized by Integrated DNA Technologies, Inc. (Munich, Germany). LightShift Chemiluminescent EMSA Kit, Biotin 3′ End DNA Labeling Kit and CL-XPosure Film were purchased from Thermo Scientific Pierce, Inc. (Rockford, USA). TransFast™ Transfection Reagent and Dual Luciferase Assay System were purchased from Promega Corp. (Madison, USA).

### Plasmid Constructs

#### Katanin-p80 promoter constructs

All deletion constructs were obtained by cloning different portions of the human genomic DNA between 2495-1496 bp upstream (excluding 5′UTR region) of the *KATNB1* start codon into pGL3 vector (Promega) (Figures 2 and 3) 1000- bp sequence (F1) was amplified by PCR using *Taq* DNA polymerase enzyme (Fermentas) from genomic DNA and cloned into Hind III/KpnI sites of pGL3 vector. Other inserts named F2, F3, F4 and F5 were obtained from F1 by using appropriate forward primers and a common reverse primer (Katnb1 R). The smallest insert F6 was obtained by hybridization of its own forward and reverse primers in a thermocycler (Table 2).

**Table 2:** Primer sequences for deletion constructs amplification.

Insert Name	Primer Name
F1 (−1374/−375) Fw	5′-AAAA**GGTACC**TCATCTGTAAATGGGAACAGGAATT-3′
F2 (−892/−375) Fw	5′-AAAA**GGTACC**ATCTGCAGGCAAAGTGAA-3′
F3 (−653/−375) Fw	5′-AAAA**GGTACC**ACACAAATTAGGTTCGAGGG-3′
F4 (−600/−375) Fw	5′-AAAA**GGTACC**GAGGCGGGACTA-3′
F5 (−534/−375) Fw	5′-AAAA**GGTACC**TTCCTTCCTCTTGATTAGTCCTATTT-3′
Katnb1 R	5′-AAAA**AAGCTT**ACCTCCGGCTCAGAGC-3′
F6 (−397/−375) Fw	5′-**GGTACC**GGGCGGGGCTCTGAGCCGGAGGT**AAGCTT**-3′
F6 Rev	5′-**AAGCTT**ACCTCCGGCTCAGAGCCCCGCCC**GGTACC**-3′

KpnI and HindIII sites are in bold. Fw: Forward primer, Rev: Reverse primer.

#### Expression constructs

The pCMV6-Elk1 (encoding amino acids 1–428 of Elk1) was used in forced experiments, transfections for qRT-PCR and Western blotting. The pRL-TK (Promega, E2241) was used as an internal control in luciferase assays.

### Bioinformatics

*KATNB1* 1000-bp promoter was determined by using UCSC Genome Browser ‘Get Genomic Sequence Near Gene’ tool (http://genome.ucsc.edu/). The promoter was analyzed for the presence of TATA-box, CAAT-box and GC-box via EBI (The European Bioinformatics Institute) ClustalW sequence analysis tool (http://www.ebi.ac.uk/Tools/msa/clustalw2/). The promoter was also analyzed for the presence of CpG island via EBI, EMBOSS CpGPlot/Report tool (http://www.ebi.ac.uk/Tools/emboss/cpgplot/). To determine deletion constructs and predict the Elk1 binding site, possible protein-DNA interactions within the promoter were analyzed using PROMO bioinformatics tool from ALGGEN server by selecting both factor’s species and site species as *Homo sapiens* with the maximum matrix dissimilarity rate 0–3% [33], [34].

### DNA Sequencing

DNA sequencing was performed by using 3100 Genetic Analyzer (Applied Biosystems) and BigDye Terminator v3.1 Cycle Sequencing kit (Applied Biosystems) according to the manufacturer’s protocol.

### Cell Culture

SH-SY5Y human neuroblastoma cells (ATCC® CRL-2266™) were used in this study as they are the prime model system for the study of neuronal functions. SH-SY5Y human neuroblastoma cells were cultured in DMEM supplemented with 10% fetal bovine serum, 2 mM L-glutamine and Penicillin/Streptomycin at a final concentration of 100 U/ml and 100 µg/ml, respectively. Cells were cultivated at 37°C in an atmosphere of 5% CO_2_. Cells were plated onto poly-L-lysine (P-2636, Sigma) coated glass coverslips at a density of 3×10^4^ cells/well for immunocytochemistry experiments; 5×10^4^ cells/well in 24-well culture dish for luciferase experiments; 1×10^6^ cells/well in 6-well plate for qRT-PCR and Western blotting experiments.

### Transient Transfection

Transfection was performed for the cells used in luciferase assay and immunocytochemistry analysis by using chemical transfection protocol. In this protocol, total of 1 µg of DNA (700 ng pGL3-F2, 200 ng pCMV6-Elk1, 100 ng *Renilla* for luciferase assay (Table 3); 1 µg pCMV6-Elk1 for immunocytochemistry experiment) was mixed with 3 µl TransFast™ Transfection Reagent in DMEM. Following removal of growth media, transfection mixture was applied to the cells and cells were incubated at 37°C for 1 h. Then, the transfection mixture was replaced with fresh growth medium and cells were cultivated for 48 h in 37°C, 5% CO_2_ incubator.

**Table 3:** Plasmids used in each transient transfection.

Construct name	Amount	Purpose
pGL3-test (F1…F6)	900 ng	Luciferase assay
pRL-TK (*Renilla*)	100 ng	Luciferase assay (internal control)
pGL3.F2	700 ng	Forced assay
pCMV6-Elk1	200 ng/1 µg	Forced assay
pCMV-Myc (empty)	200 ng/1 µg	Forced assay (for ensuring total DNA amount)

pGL3-test represents pGL3-basic vector containing one of the six different constructs (from F1 to F6).

### Luciferase Assays

*Renilla* and firefly luciferase activities were measured by using the Dual-Luciferase Reporter Assay System and Fluoroskan Ascent FL Luminometre (Thermo Electron Co., Hudson, USA). At 48 h post-transfection, growth medium was removed from cultured cells and cell lysates were obtained by adding 60 µl 1X passive lysis buffer and scraping. Cell lysates were mixed sequentially with firefly and *Renilla* specific substrates in luminometer plates according to the manufacturer’s instructions. Luminometer was programmed to perform a 2 sec pre-measurement delay while shaking the plate, followed by a 10 sec measurement period for each reporter assay. The measured activity of the firefly luciferase was normalized to that of *Renilla* luciferase. All experiments were performed in triplicates and were repeated 6 times using different DNA preparations.

### Whole Cell Extract

Whole cell extracts of Elk1 overexpressing SH-SY5Y cells, which were transfected with 5 µg pCMV6-Elk1/10^6^ cells, were prepared by using Mammalian Cell Extraction Kit (BioVision, CA, USA) according to manufacturer’s instructions.

### Recombinant Protein Production

Production of recombinant Elk1 protein which includes ETS DNA binding domain (amino acids 1–96) was performed as previously described [7]. Briefly, a 288- bp sequence of human Elk1 ETS DNA binding domain (db) was amplified and expressed as 6XHis tagged fusion protein Elk1- db. Fusion protein was purified with the help of Ni-NTA agarose method according to the manufacturers’ instruction (QIAGEN Inc., Valencia, CA, USA). Finally, purified Elk1- db was dialyzed using dialysis tubing cellulose membrane (Sigma-Aldrich Corp. St. Louis, MO, USA) and used in further EMSA analysis.

### EMSA (Electrophoretic Mobility Shift Assay)

Sequences of wild type (WT) and mutated (Mut) oligonucleotides are shown in Table 1. Oligonucleotide probes were labeled separately by using Biotin 3′ End DNA Labeling Kit that uses TdT to incorporate 1–3 biotinylated ribonucleotides on to the 3′ end of DNA strands. Labeled forward and reverse oligonucleotides were then annealed at a ratio of 1∶1 in 10 Mm Tris, 1 mM EDTA by heating to 95°C for 5 min, slow cooling by 2°C/min to their annealing temperature, annealing for 30 min and cooling to 4°C by 2°C/min (cycle number depends on Tm of the oligonucleotides). For binding reactions, ∼2 µg whole extracts of SH-SY5Y cells or Elk1- db protein were incubated with 20 fmol biotinylated oligonucleotides in binding buffer (pH 7.5), including 10 mM Tris, 50 mM KCl, 1 mM dithiothreitol (DTT), 1 µg Poly (dI·dC), 5% glycerol, 1 mM EDTA, 0.3% bovine serum albumin (BSA) and 1X Protease Inhibitor Cocktail for 20 min at room temperature. 1000-fold molar excess of unlabeled competitor oligonucleotides were also used in binding reaction. Complexes and free DNAs were resolved on a 5–8% nondenaturating polyacrylamide gel in 0.5 X TBE by electrophoresis for 1 h at 120 V at 4°C. The separated bands on the gel were then transferred to Biodyne A Nylon Membranes (Pierce, Rockford, USA) by using Trans-Blot® SD (Bio-Rad, California, USA) at 20 V for 30 min at 4°C. Cross-link transfer of DNA to membrane was achieved by incubating the membrane with 254 nm UV bulbs for 12 min. In order to detect biotin-labeled DNA, Chemiluminescent Nucleic Acid Detection Module Kit (Pierce, Rockford, USA) was used. The membrane was exposed to X-ray film for 2 min and then, was developed in Kodak Medical X-ray Processor according to manufacturer’s instruction. In super-shift assays, 1 µg of Tetra-His antibody (QIAGEN Inc., CA, USA) was added prior to the addition of 150 ng Elk1- db protein.

### Total RNA Extraction and cDNA Synthesis

1×10^6^ cells were transfected with 2 µg of DNA (pCMV6-Elk1) by using Amaxa Nucleofector (Lonza Ltd., Basel, Switzerland) and Nucleofector® Kit V reagent according to the manufacturer’s protocols. After 24 h of nucleofection, the cells were prepared for isolation of RNA. Total RNA was extracted from Elk1 transfected SH-SY5Y cells by using High Pure RNA Isolation Kit (Roche) in accordance with the manufacturer’s instructions. 1 µg of total RNA was reverse transcribed by using Random Hexamer primers and Reverse Transcriptase (Roche) according to the manufacturer’s instructions.

### Quantitative Real Time – PCR

Specific katanin-p80 probe and primers were purchased from Universal Probe Library (Roche Applied Science). Beta-actin (Roche Universal Probe Library Human ACTB Gene Assay) gene was used as reference for relative expression analysis. Quantitative Real Time – PCR (qRT-PCR) reactions were performed with Light Cycler® 480 Probes Master qRT-PCR Kit using a Roche Light Cycler 480 according to the following program: initial denaturation at 95°C for 10 min for 1 cycle, denaturation at 95°C for 10 sec, amplification at 60°C for 30 sec, extension at 72°C for 1 sec for 45 cycles. ΔΔCt method was used to analyze qRT-PCR data. According to Schmittgen et al. [35], reaction efficiency rate between 1.8–2.2 and error rate below 0.2 allows the use of this method. Therefore, the reaction efficiency and error rate values were determined for each gene and all of them were in expected ranges. The method uses the equation 2- ΔΔCT = [(CT gene of interest - CT internal control) treated sample - (CT gene of interest - CT internal control) control sample)] to calculate and present data as ‘fold change’ in expression.

### KCl Treatment

To increase SUMOylation [22] in desired experimental conditions, following 24 h of transfection, cells were depolarized by KCl. KCl treatment was performed for 1 h in the growth medium containing 31% depolarization buffer (170 mM KCl, 2 mM CaCl2, 1 mM MgCl2, 10 mM HEPES) at a final concentration of 50 mM KCl.

### Immunocytochemistry

SH-SY5Y cells were plated at a density of 3×10^4^ cells/well on poly-L-lysine coated coverslips. Cells were then transfected with Elk1 expression vectors using TransFast™ Transfection Reagent according to the manufacturer’s instructions. Naive cells were used as negative control for examining endogenous katanin-p80 level. After 24 h of transfection, one group of Elk1 transfected cells was treated with KCl for 1 h to increase SUMO conjugation [22]. After 48 h, all the cells were fixed with absolute methanol solution for 15 min at −20°C. Following fixation, cells were washed with 1X phosphate-buffered saline 3 times for 5 min and blocked with blocking buffers (10% donkey serum, 10 mg/mL bovine serum albumin in phosphate-buffered saline) for 1 h. Then, the cells were incubated with goat polyclonal anti-katanin-p80 antibody in 1∶500 dilutions overnight at +4°C. Next day, cells were incubated with Alexa Fluor 488 donkey anti-goat secondary antibody (Invitrogen Corp.) in 1∶1000 dilutions for 1 h at 37°C in dark. Coverslips were then mounted on Mounting Medium (Sigma-Aldrich Corp.) and analyzed with Leica TCS SP2 SE Confocal Microscope (Buffalo Grove, IL, USA). In order to determine the expressional values of the target proteins, pixel analysis was used from raw confocal immunocytochemistry images.

### Western Blotting

1×10^6^ cells were transfected with 2 µg of DNA (pCMV6-Elk1) by nucleofection. After 24 h, total protein was extracted from cells. Equal amounts (2 µg protein/lane) of proteins obtained from whole cell extracts of untransfected and Elk1 transfected samples were separated by SDS-PAGE and transferred onto nitrocellulose membrane. Membrane was blocked in 5% skim milk powder/TBST (TBS containing 0.1% Tween 20) for 2 h at room temperature, and then stained with the following primary antibodies at indicated dilutions overnight at 4°C; rabbit polyclonal anti-Elk1 antibody (1∶500 Cell Signaling), rabbit polyclonal anti-katanin-p80 antibody (1∶500, Sigma) and rabbit monoclonal anti-β-actin (1∶1000, Cell Signaling) in 5% skim milk powder/TBST. Membrane was then incubated with HRP conjugated goat anti-rabbit IgG secondary antibody (1∶5000, Promega) for 2 h at room temperature. Bands were visualized using Super Signal West Femto Chemiluminescent Substrate (Thermo Scientific) and Kodak Medical X-ray Processor.

### Immunoprecipitation

Total proteins obtained from whole cell extracts were used in immunoprecipitation experiments. Firstly, rabbit polyclonal anti-Elk1 antibody (Cell Signaling) was diluted in 1∶50 µg/ µl in PBS-T buffer and incubated with 50 µl Dynabeads Protein G (Invitrogen) for 10 min according to manufacturer’s protocol. Then, cross-linked beads were incubated with 1 mg total protein for 15 min at room temperature. Unbound proteins were washed 3 times with 200 µl PBS and bound proteins were eluted using 20 µl 50 mM Glycine buffer (pH 2.8). Eluted proteins were finally incubated for 3 min at 70°C in order to dissociate the complex. SUMOylation level of immunoprecipitated Elk1 was detected by Western blotting and rabbit monoclonal anti-SUMO-1 antibody (1∶500, Cell Signaling) and rabbit polyclonal anti-SUMO-2/3 antibody (1∶500, Santa Cruz) were used as primary antibody.

### Integrative Pixel Analysis

Photoshop CS5 Software was used to analyze the relative intensity of the protein bands in Western blot images. The intensity value for each protein band was calculated by measuring the selected band area and pixel values. Obtained values were normalized against values of internal control. Immunocytochemistry analysis was also performed with the same software. Total fluorescence per cell and corrected integrated density for each cell were obtained by subtracting background from raw images which were taken under same microscopy settings.

### Statistical Analysis

All the experiments were statistically analyzed by two-tailed unpaired student’s t test using GraphPad Instat 3 software. Error bars in the graphs were generated using ± SD values. P value under 0.05 was considered significant for all statistical analysis.

## References

[1] McNally FJ, Vale RD (1993) Identification of katanin, an ATPase that severs and disassembles stable microtubules. Cell 75: 419–429. 8221885 10.1016/0092-8674(93)90377-3

[2] Hazan J, Fonknechten N, Mavel D, Paternotte C, Samson D, et al (1999) Spastin, a new AAA protein, is altered in the most frequent form of autosomal dominant spastic paraplegia. Nature Genetics 23(3): 296–303. 10610178 10.1038/15472

[3] McNally KP, Bazirgan OA, McNally FJ (2000) Two domains of p80 katanin regulate microtubule severing and spindle pole targeting by p60 katanin. Journal of Cell Science 113: 1623–1633. 10751153 10.1242/jcs.113.9.1623

[4] Hartman JJ, Mahr J, McNally K, Okawa K, Iwamatsu A, et al (1998) Katanin, a microtubule-severing protein, is a novel AAA ATPase that targets to the centrosome using a WD40-containing subunit. Cell 93: 277–287. 9568719 10.1016/s0092-8674(00)81578-0

[5] Ahmad FJ, Yu W, McNally FJ, Baas PW (1999) An Essential Role for Katanin in Severing Microtubules in the Neuron. The Journal of Cell Biology 145: 305–315. 10209026 10.1083/jcb.145.2.305 PMC2133110

[6] Yu W, Solowska JM, Qiang L, Karabay A, Baird D, et al (2005) Regulation of microtubule severing by katanin subunits during neuronal development. Journal of Neuroscience 25 (23): 5573–5583. 10.1523/JNEUROSCI.0834-05.2005 PMC1201504 15944385

[7] Canbaz D, Kırımtay K, Karaca E, Karabay A (2011) SPG4 gene promoter regulation via Elk1 transcription factor. Journal of Neurochemistry 117: 724–734. 21395583 10.1111/j.1471-4159.2011.07243.x

[8] Yu W, Qiang L, Solowska JM, Karabay A, Korulu S, et al (2008) The microtubule-severing proteins spastin and katanin participate differently in the formation of axonal branches. Molecular Biology of the Cell 19(4): 1485–1498. 18234839 10.1091/mbc.E07-09-0878 PMC2291400

[9] Sgambato V, Vanhoutte P, Pages C, Rogard M, Hipskind R, et al (1998) In vivo expression and regulation of Elk-1, a target of the extracellular-regulated kinase signaling pathway, in the adult rat brain. Journal of Neuroscience 18: 214–226. 9412502 10.1523/JNEUROSCI.18-01-00214.1998 PMC6793414

[10] Pastorcic M, Das HK (2003) Ets transcription factors ER81 and Elk1 regulate the transcription of the human presenilin 1 gene promoter. Molecular Brain Research 113: 57–66. 12750007 10.1016/s0169-328x(03)00090-1

[11] Demir O, Korulu S, Yildiz A, Karabay A, Aksan-Kurnaz I (2008) Elk-1 interacts with neuronal microtubules and relocalizes to the nucleus upon phosphorylation. Molecular and Celular Neuroscience 40: 111–119. 10.1016/j.mcn.2008.10.004 19013529

[12] Buchwalter G, Gross C, Wasylyk B (2004) Ets ternary complex transcription factors. Gene 324: 1–14. 14693367 10.1016/j.gene.2003.09.028

[13] Yordy JS, Muise-Helmericks RC (2000) Signal transduction and the Ets family of transcription factors. Oncogene 19: 6503–6513. 11175366 10.1038/sj.onc.1204036

[14] Yang S, Jaffray E, Hay RT, Sharrocks AD (2003) Dynamic Interplay of the SUMO and ERK Pathways in Regulating Elk-1 Transcriptional Activity. Molecular Cell 12: 63–74. 12887893 10.1016/s1097-2765(03)00265-x

[15] Sherwood NT, Sun Q, Xue M, Zhang B, Zinn K (2004) Drosophila spastin regulates synaptic microtubule networks and is required for normal motor function. PLoS Biology 2(12): e429. 15562320 10.1371/journal.pbio.0020429 PMC532392

[16] Gillingwater TH, Wishart TM (2013) Mechanisms underlying synaptic vulnerability and degeneration in neurodegenerative disease. Neuropathology and Applied Neurobiology doi: 10.1111/nan.12014. 10.1111/nan.12014 23289367

[17] Gozes I (2011) Microtubules (tau) as an emerging therapeutic target: NAP (davunetide). Current Pharmaceutical Design 17(31): 3413–7. 21902667 10.2174/138161211798072553

[18] Henson BJ, Zhu W, Hardaway K, Wetzel JL, Stefan M, et al (2012) Transcriptional and post-transcriptional regulation of SPAST, the gene most frequently mutated in hereditary spastic paraplegia. PLoS One 7(5): e36505. 22574173 10.1371/journal.pone.0036505 PMC3344893

[19] Daftary GS, Tetrault AM, Jorgensen EM, Sarno J, Taylor HS (2011) A novel role for the AAA ATPase spastin as a HOXA10 transcriptional corepressor in Ishikawa endometrial cells. Molecular Endocrinology 25(9): 1539–49. 21757506 10.1210/me.2011-0001 PMC3165913

[20] Ye B, Kim JH, Yang L, McLachlan I, Younger S, et al (2011) Differential regulation of dendritic and axonal development by the novel Krüppel-like factor Dar1. Journal of Neuroscience 31(9): 3309–19. 21368042 10.1523/JNEUROSCI.6307-10.2011 PMC3086283

[21] Mancuso G, Rugarli EI (2008) A cryptic promoter in the first exon of the SPG4 gene directs the synthesis of the 60-kDa spastin isoform. BMC Biology 6: 31. 18613979 10.1186/1741-7007-6-31 PMC2474578

[22] Lu H, Liu B, You S, Xue Q, Zhang F, et al (2009) The activity-dependent stimuli increase SUMO modification in SHSY5Y cells. Biochemical and Biophysical Research Communications 390: 872–876. 19840774 10.1016/j.bbrc.2009.10.065

[23] Ross S, Tienhaara A, Lee MS, Tsai LH, Gill G (2002) GC box-binding transcription factors control the neuronal specific transcription of the cyclin-dependent kinase 5 regulator p35. The Journal of Biological Chemistry 277(6): 4455–4464. 11724806 10.1074/jbc.M110771200

[24] Kim KI, Baek SH, Chung CH (2002) Versatile protein tag, SUMO: its enzymology and biological function. Journal of Cellular Physiology 191: 257–268. 12012321 10.1002/jcp.10100

[25] Sandelin A, Carninci P, Lenhard B, Ponjavic J, Hayashizaki Y, et al (2007) Mammalian RNA polymerase II core promoters: insights from genome-wide studies. Nature Review Genetics 8: 424–436. 10.1038/nrg2026 17486122

[26] Zolova OE, Wight PA (2011) YY1 negatively regulates mouse myelin proteolipid protein (Plp1) gene expression in oligodendroglial cells. American Society for Neurochemistry 3(4): 223–232. 10.1042/AN20110021 PMC3207217 21973168

[27] Morshed M, Ando M, Yamamoto J, Hotta A, Kaneoka H, et al (2006) YY1 binds to regulatory element of chicken lysozyme and ovalbumin promoters. Cytotechnology 52(3): 159–70. 19002874 10.1007/s10616-006-9017-4 PMC3449407

[28] Sui G, Affar el B, Shi Y, Brignone C, Wall NR, et al (2004) Yin Yang 1 is a negative regulator of p53. Cell 117(7): 859–72. 15210108 10.1016/j.cell.2004.06.004

[29] Bohren KM, Nadkarni V, Song JH, Gabbay KH, Owerbach DD (2004) A M55V polymorphism in a novel SUMO gene (SUMO-4) differentially activates heat shock transcription factors and is associated with susceptibility to type I diabetes mellitus. The Journal of Biological Chemistry 279: 27233–27238. 15123604 10.1074/jbc.M402273200

[30] Yang SH, Vickers E, Brehm A, Kouzarides T, Sharrocks AD (2001) Temporal recruitment of the mSin3A-histone deacetylase corepressor complex to the ETS domain transcription factor Elk-1. Molecular and Cellular Biology 21(8): 2802–2814. 11283259 10.1128/MCB.21.8.2802-2814.2001 PMC86910

[31] Yang SH, Sharrocks AD (2004) SUMO promotes HDAC-mediated transcriptional repression. Molecular Cell 13(4): 611–617. 14992729 10.1016/s1097-2765(04)00060-7

[32] Cenik C, Derti A, Mellor JC, Berriz GF, Roth FP (2010) Genome-wide functional analysis of human 5′ untranslated region introns. Genome Biology 11(3): R29. 20222956 10.1186/gb-2010-11-3-r29 PMC2864569

[33] Messeguer X, Escudero R, Farré D, Núñez O, Martínez J, et al (2002) PROMO: detection of known transcription regulatory elements usings pecies-tailored searches. Bioinformatics 18(2): 333–336. 11847087 10.1093/bioinformatics/18.2.333

[34] Farré D, Roset R, Huerta M, Adsuara JE, Roselló L, et al (2003) Identification of patterns in biological sequences at the ALGGEN server: PROMO and MALGEN. Nucleic Acids Research 31(13): 3651–3653. 12824386 10.1093/nar/gkg605 PMC169011

[35] Schmittgen TD, Livak KJ (2008) Analyzing real-time PCR data by the comparative C (T) method. Nature Protocols 3: 1101–1108. 18546601 10.1038/nprot.2008.73

